# Variations in diabetes remission rates after bariatric surgery in Spanish adults according to the use of different diagnostic criteria for diabetes

**DOI:** 10.1186/s12902-017-0201-7

**Published:** 2017-08-15

**Authors:** María R. Alhambra-Expósito, María J. Molina-Puerta, María I. Prior-Sánchez, Gregorio Manzano-García, Alfonso Calañas-Continente, María A. Gálvez-Moreno

**Affiliations:** UGC Endocrinología y Nutrición, Hospital Reina Sofía, Avenida Menéndez Pidal, s/n, 14004 Córdoba, Spain

**Keywords:** Bariatric surgery, Diabetes, Obesity, Gastric bypass

## Abstract

**Background:**

There are multiple criteria to define remission of type 2 diabetes (DM2) after bariatric surgery but there is not a specific one widely accepted. Our objectives were to compare diagnostic criteria for DM2 remission after bariatric surgery: Criteria from Spanish scientific associations (SEEN/SEEDO/SED) and from the American Diabetes Association (ADA). We also aim to analyse the degree of correlation between these sets of criteria.

**Methods:**

Retrospective observational study in 127 patients undergoing bariatric surgery in a single centre (Hospital Universitario Reina Sofía, Córdoba, Spain) between January 2001 and December 2009. We analysed DM2 remission following bariatric surgery comparing DM2 diagnostic criteria approved by Spanish scientific associations and ADA criteria.

**Results:**

In total, 62.2% of patients were women; mean age was 47.1 years. Following surgery, 52% achieved complete remission according to ADA criteria, and 63.8% following the criteria approved by Spanish associations (*p* = 0.001);18.9 and 8.7%, respectively, showed partial remission (*p* = 0.007), and 29.1 and 27.6% no remission, according to the criteria approved by each association (*p* = 0.003). There was good correlation between both sets of criteria (Rho 0.781; *p* < 0.001).

**Conclusions:**

In our series, using more stringent criteria for defining DM2 remission (ADA criteria) results in a lower rate of remission, although we found a a high degree of correlation between both sets of criteria.

## Background

Worldwide prevalence of type 2 diabetes (DM2) continues to increase simultaneously with obesity rates [1, 2]. Over 60% of DM2 patients are obese [1], and this tandem is now a public health problem. Recent studies have shown that medical therapy for DM2 and comorbid obesity is not as successful as bariatric surgery, suggesting that modest weight loss and DM2 control is hard to achieve [3, 4]. American Diabetes Association (ADA) recommended for the first time in their 2010 edition of their standards of care [5] to consider bariatric surgery in the treatment of DM2 patients with a body mass index (BMI) ≥ 35 kg/m^2^. This indication is now widely accepted by leading international associations [6–9], particularly when DM2 or comorbidities are refractory to life style changes and pharmacological treatment.

The choice of criteria to define remission of DM2 after bariatric surgery is still widely debated. Some authors consider the withdrawal of medication to be the best criteria [10], while others suggest using various fasting plasma glucose (FPG) and glycated haemoglobin (HbA1c) cut-off points [11–13], or a combination of both [14–16]. After publication of the meta-analysis by Buchwald et al. in 2004, DM2 remission criteria recommended by these authors (HbA1c <6% and FPG 100 mg/dl) were the most used [17].

However, a consensus group from the ADA (Buse’s consensus group [18]), consisting of experts in endocrinology, diabetes education, bariatric surgery and haematology-oncology, proposed in 2009 a new, far more stringent standard set of definition criteria based on biochemical (HbA1c and FPG levels) and clinical parameters (need for pharmacological treatment and duration of remission). In 2013, scientific associations in Spain published a position statement on metabolic surgery in patients with DM2 stating their set of criteria for define DM2 remission, which were similar to those approved by the ADA consensus group [19]. Considering the difficulty in establishing remission of DM2, Sánchez- Pernaute and Scopinaro have proposed using the ADA HbA1c cut-off point to diagnose DM2 [11, 20].

The aim of this study is to compare DM2 remission rates 5 years after bariatric surgery using the criteria approved by Spanish associations and ADA diagnostic criteria for DM2.

## Methods

We conducted a retrospective observational study in 127 patients undergoing bariatric surgery in a single centre (Hospital Universitario Reina Sofía, Córdoba, Spain) between January 2001 and December 2009. All patients were diagnosed with DM2 and obesity (BMI ≥ 35 kg/m^2^) before surgery, and had at least 3 years of follow-up with documentation of FPG, HbA1c levels, and body weight. Three types of bariatric surgery were performed: roux-en-Y gastric bypass, sleeve gastrectomy and gastric band. The type of surgery was chosen depending on the patient’s preoperative characteristics. Those undergoing bariatric surgery with a different technique were also excluded because of their low incidence. Patients who underwent a second bariatric surgery (including conversion, revisional and reversal procedures) were also excluded.

All procedures in studies involving human participants were performed in accordance with the ethical standards of the institutional review board of Hospital Universitario Reina Sofía and with the principles of the Declaration of Helsinki 2013. For this type of study formal consent was not required. This study was approved by the Ethics Committee of the Hospital Universitario Reina Sofía.

Demographic (age and sex), anthropometric (weight, height, BMI) and analytical (FPG and HbA1c) variables were collected during follow-up.

Remission of DM2 was defined using 2 different criteria: (1) those approved by Spanish scientific associations, based on HbA1c and FPG cut-off levels and need for diabetes medication, which classify their status as complete, prolonged or partial remission, improvement, or no remission, as shown in Table 1 [19]; and (2) ADA criteria, based on HbA1c levels and need for diabetes medication, which classify patients’ status as complete or partial remission, optimal control, or no remission, as shown in Table 2 [20].

**Table 1:** Post-bariatric surgery DM2 remission criteria approved by Spanish scientific associations [11, 12]

Complete remission	HbA1c < 6.5%, FPG < 100 mg/dl [5.6 mmol/l] and no need for diabetic medication for at least 1 year of follow-up
Prolonged remission	Complete remission for more than 5 years.
Partial remission	HbA1c < 6.5%, FPG: 100-125 mg/dl [5.6-6.9 mmol/l] and no need for diabetic medication for at least 1 year of follow-up
Improvement	HbA1c < 7% with diabetic medication.
No remission	HbA1c ≥ 6.5%, FPG ≥ 126 [6.9 mm/L] and/or need for diabetic medication.

*DM2* type 2 diabetes mellitus, *FPG* fasting plasma glucose, *HbA1c* glycated haemoglobin

**Table 2:** Simplified DM2 diagnostic criteria based on HbA1c proposed by ADA [13]

Complete remission	HbA1c < 5.7%, with no need for diabetes medication for at least 1 year of follow-up
Partial remission	HbA1c 5.7–6.4%, with no need for diabetes medication for at least 1 year of follow-up
Optimal control	HbA1c < 7% with or without diabetes medication.
No remission	HbA1c > 6.5% or active hypoglycaemic treatment

*ADA* American Diabetes Association, *DM2* type 2 diabetes mellitus, *HbA1c* glycated haemoglobin

For the sake of statistical analysis, the complete and prolonged remission groups defined by the Spanish associations were included in the same group, given that prolonged remission according to the Spanish associations’ criteria is included in the criteria for complete remission. Complete and partial remission and no remission criteria from both classification systems were compared. The improvement and optimal control criteria from the Spanish and ADA systems, respectively, were not compared separately, as these were both included in their respective no-remission classification.

Excess weight loss percentage (EWL%) was calculated using the formula: [(initial weight- follow-up weight)/(initial weight – ideal weight)] X 100. Ideal weight was calculated for a BMI of 21 kg/m^2^in women, and 23 kg/m^2^ in men. DM2 was diagnosed according to ADA criteria: FPG > 126 mg/dl, HbA1c ≥ 6.5%, random blood glucose ≥200 mg/dl, or use of insulin or oral diabetes medication.

Glucose was measured in mg/dl, and HbA1c results are reported in NGSP/DCCT (%) units (to 1 decimal point) [21].

### Statistical analysis

In the descriptive analysis, qualitative variables in each category are expressed as absolute frequencies and percentage. Quantitative variables are expressed with their mean ± standard deviation (SD) using the Shapiro-Wilk to test for normality. Non-parametric tests were used for variables with non-normal distribution, depending on the characteristics of the parameter. The chi-square analysis was used to test the association between qualitative variables. Student’s t-test (normal distribution) or Mann-Whitney U test (non-normal distribution) were used to compare means; ANOVA was used to compare means between non-dichotomous variables. Paired qualitative variables were compared using McNemar’s test. Spearman’s correlation coefficient was used as a measure of correlation between non-parametric variables. Statistical significance was set at 5%, and statistical analysis was performed using SPSS version 15.0 for Windows.

## Results

**Fig. 1:**
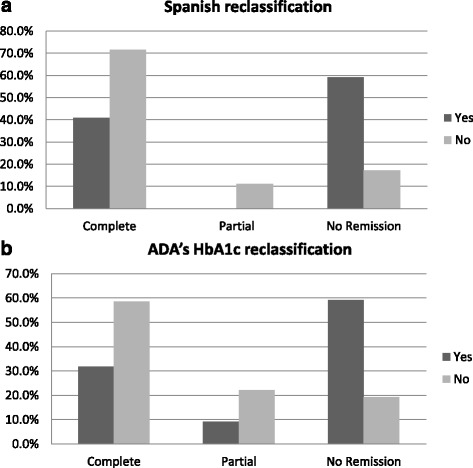
Remission rate in patients using or no using insulin. **a** Using Spanish reclassification; **b** Using ADA HbA1c reclassification

A total of 127 patients (62.2% women) with DM2 were included in the study. Baseline and postoperative characteristics of patients are shown in Table 3. Mean age at the time of surgery was 47 ± 8 years; mean preoperative BMI was 50 ± 7 kg/m^2^, FPG was 134 ± 53 mg/dl and HbA1c was 8 ± 7%. As shown in Table 3, patients presenting complete remission are younger, with lower postoperative BMI, and lower pre- and postoperative FPG and HbA1c levels. These intra-group differences were found in both classifications (ANOVA test). Patients with no remission using both reclassification systems were more likely to have been on insulin (Fig. 1). Roux-en-Y gastric bypass was performed in 96% of patients, sleeve gastrectomy in 3%, and gastric band in the remaining 1%. Before surgery, 39 patients were in treatment with metformin, 6 with insulin, and 13 with other oral diabetes medication (ODM). Nineteen (19) patients were in treatment with a combination of metformin and insulin, and 14 with metformin and other ODM. Of the remaining patients, 36 were following a dietary and exercise programme, as DM2 had been diagnosed less than 6 months before surgery. After a mean follow-up of 5 ± 2 years, mean BMI, FPG and HbA1c were 37 ± 6 kg/m^2^, 100 ± 28 mg/dl and 6 ± 1%, respectively (Table 3). The difference between these and variables before and after surgery was statistically significant (paired samples t-tests; *p* = 0.001, *p* = 0.007 and *p* = 0.003, respectively).

**Table 3:** Patient characteristics and diabetes remission, grouped by different definition criteria

	Total	SEEN/SEEDO/SED				ADA			
		Complete	Partial	No Remission	*P* value*	Complete	Partial	No Remission	*P* value*
No. Patients	127	81	11	35	–	66	24	37	–
Age (years)	47.1 ± 8.5	45.1 ± 8.8	48.8 ± 6.3	51.2 ± 6.8	**0.001**	45.1 ± 8.1	45.6 ± 8.8	51.5 ± 6.8	**0.001**
Women	62	55	1	23	0.241	41	13	25	0.142
Pre BMI (kg/m^2^)	50.9 ± 7.6	51.9 ± 8.1	49.9 ± 2.7	49.0 ± 6.9	0.056	51.5 ± 7.6	51.9 ± 8.2	49.2 ± 6.8	0.319
Post BMI (kg/m^2^)	37.2 ± 6.1	36.5 ± 6.4	38.4 ± 3.5	38.5 ± 6.1	**0.046**	35.8 ± 5.4	38.9 ± 7.6	38.5 ± 5.9	**0.030**
Pre FPG (mg/dl)	134.8 ± 53.7	115.7 ± 37.9	159.2 ± 61.4	155.8 ± 63.1	**0.001**	115.3 ± 34.5	150.7 ± 70.8	154.6 ± 16.5	**0.000**
Post FPG (mg/dl)	100.0 ± 28.5	85.1 ± 121.8	111.6 ± 16.1	126.0 ± 34.1	**0.002**	87.7 ± 15.0	87.5 ± 12.8	126.6 ± 33.1	**0.003**
Pre HbA1c (%)	8.0 ± 7.6	7.6 ± 1.9	7.9 ± 1.6	9.0 ± 2.0	**0.000**	7.6 ± 1.9	7.8 ± 16	9.0 ± 2.0	**0.000**
Post HbA1c (%)	5.8 ± 0.8	5.5 ± 0.4	5.5 ± 0.4	6.7 ± 0.9	**0.018**	5.3 ± 0.3	6.0 ± 0.2	6.8 ± 0.9	**0.001**
EWL%	51.0 ± 18.3	55.4 ± 17.5	40.9 ± 12.3	43.9 ± 18.7	0.573	54.8 ± 16.8	50.9 ± 20.1	44.2 ± 18.3	**0.001**

The bold numbers remark the differences

*Intra-group differences in both classifications: Anova test

*ADA* American Diabetes Association. *EWL%* excess weight loss percentage, *Post BMI* postoperative body mass index, *Pre BMI*: preoperative body mass index, *Post FPG* postoperative fasting plasma glucose, *Pre FPG* preoperative fasting plasma glucose, *Post HbA1c* postoperative glycated haemoglobin, *Pre HbA1c* preoperative glycated haemoglobin, *SEEN /SEEDO /SED* Sociedad Española de Endocrinología y Nutrición / Sociedad Española para el Estudio de la Obesidad / Sociedad Española de Diabetes

Following surgery, according to simplified HbA1c criteria, 52% achieved remission, 18.9% improvement, and 29.1% no remission. According to Spanish criteria, 63.8% of patients presented with complete remission of DM2 (33.1% with prolonged remission), 8.7% achieved partial remission, and 27.6% no remission (of which 21.3% showed improvement of DM2). Statistically significant differences were observed between these reclassifications (McNemar’s test; *p* < 0.001). The reason behind this was that of 81 patients showing complete remission with the Spanish criteria, 59 achieved complete remission, 21 out of those 81 showed partial remission and 1 patient was classified as non remitter according to ADA criteria. Thirty-five (35) patients classified as non-remitters according to the Spanish reclassification were also considered non-remitters with the ADA criteria. Other 2 patients classified as non-remitters with ADA criteria were categorized in the group of complete remission (1) and partial remission (1) by Spanish reclassification.

There is a good correlation between the reclassification systems (Spearman’s rho = 0.781, *p* < 0.001). Fifty-nine (59) patients showed complete remission, 3 partial remission, and 35 no remission under both sets of criteria (Table 4).

**Table 4:** Postoperative diagnostic reclassification

		Spanish reclassification			Total
		Complete remission	Partial remission	No remission	
ADA HbA1c reclassification	Complete remission	59	7	0	66
	Partial remission	21	3	0	24
	No remission	1	1	35	37
Total		81	11	35	127

*ADA* American Diabetes Association, *HbA1c* glycated haemoglobin

## Discussion

There is a certain amount of controversy regarding the best criteria to define diabetes remission following bariatric surgery. EWL% is known to be a predictive factor of DM2 remission: the higher the EWL%, the greater the likelihood of remission [22]. This was found to be true in our series. Between the partial remission and no remission groups according to the Spanish criteria, although there were differences, they were not statistically significant. Remission is also associated with age and the degree of DM2 control. Our study has shown that complete DM2 remission is more common in young patients and in those with better-controlled mean HbA1c and FPG levels. Our findings also show that more stringent criteria for defining DM2 remission results in a lower rate of complete remission categorised as total remission. Instead, they would be categorised as partial remission (63.8% with complete remission under Spanish criteria vs. 52% complete remissions following the ADA’s more stringent criteria [HbA1c < 5.7%]). Some authors consider DM2 to be in remission purely on the basis of clinical criteria (no need for diabetes medication) or a single analytical finding: FPG or HbA1c [2, 23]. In the meta-analysis performed by Buchwald et al. [24], remission rates varied considerably (54.9-95.1%), depending on the type of surgery or definition criteria used (FPG < 100 mg/dl or HbA1c < 6%). Other studies and systematic reviews with high remission rates also used less demanding criteria [2, 23] and relatively short (1 to 2 years) follow-up periods. Different approaches to define remission have also led to false expectations with regard to the true percentage of patients achieving long-term diabetes remission. For example in the SOS study the 2-year DM2 remission rate of 72% declined to a 36% remission rate after 10 years [14]. In our series, after a mean follow-up of 64 months, 52% of patients achieved complete remission under ADA criteria, i.e. HbA1c < 5.7% and no need for diabetes medication. This is consistent with other studies [25–27] reporting remission rates of 40.6, 43.6 and 50%. Specifically, Ramos-Levi [27] et al., in a retrospective cohort of 110 patients, compared DM2 remission rates based on the same definition criteria used in our study. There were some differences between these studies; though mean BMI in their patients was lower than in our study (43.6 ± 5.5 vs 50.9 ± 7.6 kg/m2) and the percentage of patients on insulin in their study was higher than in our study (44.5% vs 19.7%). They found no statistically significant differences between remission rates (50% of total remission using both the Spanish and ADA criteria). However, follow-up was limited to 18 months. In our 5-year follow-up study, we found that remission rates did differ depending on the definition criteria used. This is probably due to the fact that our follow-up period is one of the longest of all studies in DM2 remission. This begs the question whether these remission rates can be sustained over a long-term follow-up of 10 years or more. ADA remission criteria are the most stringent and the most widely used. To confirm DM2 remission, therefore, these same criteria should be applied and updated according to established standards.

One limitation of our study lies in the fact that retrospective cohorts are more susceptible to bias, such as loss to follow-up. In addition, some relatively important data, such as time from onset of diabetes to bariatric surgery, were missing. Another possible limitation is that we did not take into consideration differences between surgical techniques, because very few sleeve gastrectomies were performed during the study period due to the preferences and experience of the surgical team. Into the bargain we cannot know whether patients with new diabetes drugs such as SGLT-2 inhibitors or GLP-1 analogues would have similar or unrelated remission rates, because during the years in which these patients (2001-2009) were included, these drugs were not available in Spain.

## Conclusion

In conclusion, we believe that strict criteria based on those established by the ADA with the addition of the Spanish prolonged remission criteria should be used to determine true remission of diabetes following bariatric surgery. Further studies in larger cohorts with longer follow-up periods are needed to conclusively show the best criteria for defining post-bariatric surgery diabetes remission.

## Abbreviations

ADA: American Diabetes Association
BMI: Body mass index
DM2: Type 2 diabetes
EWL%: Excess weight loss percentage
FPG: Fasting plasma glucose
HbA1c: Glycated haemoglobin
SD: Standard deviation
SEEN/SEEDO/SED: Sociedad Española de Endocrinología /Sociedad Española para el estudio de la obesidad / Sociedad Española de Diabetes.
